# The Pan-African Society of Cardiology (PASCAR) in 2013 and beyond

**Published:** 2013-06

**Authors:** Anastase Dzudie, Bongani M Mayosi

**Affiliations:** Department of Internal Medicine, Douala General Hospital, Cameroon; Department of Medicine, Groote Schuur Hospital and University of Cape Town, Cape Town, South Africa

## Abstract

**Abstract:**

The biennial Congress of the Pan-African Society of Cardiology (PASCAR) was held in Dakar from 16 to 19 May 2013 under the patronage of his Excellency, Macky Sall, president of the Republic of Senegal. This meeting was remarkable in the diversity of its 700 participants from English-, French- and Portuguese-speaking Africa. Important aspects of cardiovascular disease in Africa were presented in 195 abstracts and numerous talks; the topics were hypertension, obesity, diabetes, heart failure, cardiomyopathies, coronary heart disease, stroke and rheumatic heart disease. The general assembly meeting was marked by the review and adoption of a new constitution and elections of a new PASCAR governing council that will be in office for the next four years. The new leadership of PASCAR has committed itself to strengthening the administrative infrastructure of the organisation, developing programmes to address education and training needs of African cardiovascular practitioners, developing a pan-African multi-national research platform, and ensuring that ministries of health implement national programmes for the prevention and control of cardiovascular and other non-communicable diseases.

## Introduction

Since its conception by a small group of African cardiologists during the late 1970s, the Pan-African Society of Cardiology (PASCAR) has undergone three phases. These include an early growth spurt, characterised by regular continental meetings from 1981 to 1997; a period of stagnation from 1998 to 2003, during which no PASCAR meetings were held; and the renaissance phase that started in Accra, Ghana in October 2004 at a conference that was attended by 40 delegates, mainly from Cameroon, Ghana, Nigeria and South Africa.^1^ Since Ghana, PASCAR has been gaining momentum in its work of galvanising health practitioners in Africa to improve clinical care and prevention of cardiovascular disease.

In 2007, the 8th PASCAR congress held in Nairobi, Kenya attracted over 300 participants from countries in all regions of Africa and beyond. A similarly global audience attended the 9th PASCAR congress in Abuja, Nigeria in September 2009, while the 10th congress two years later in Kampala, Uganda attracted about 400 global participants, mainly from English-speaking Africa. The achievement of the 2013 Dakar meeting was to bring together English-, French- and Portuguese-speaking Africans and foster understanding between these historically separated groups.

## Opening ceremony

The PASCAR congress was held in Dakar from Wednesday 15 to Sunday 19 May 2013 under the patronage of his Excellency Macky Sall, president of the Republic of Senegal. At the opening ceremony, one minute of silence was observed in honour of the three distinguished members of PASCAR who have passed away since the previous meeting (the late President of PASCAR, Prof Oluwole Adebo of Nigeria, the late Editor-in-Chief of the *Cardiovascular Journal of Africa*, Prof Andries Brink of South Africa, and Prof Ndobo Pierre from Cameroon).

Prof Walinjon Muna, a past president of PASCAR, gave the opening address on the burden of cardiovascular diseases (CVD) in Africa and how to define specific strategies to control this rising burden of disease. He was followed by Prof Abdou Ba, president of the organising committee, who expressed his gratitude to all contributors, with particular credit to the highest Senegalese authority for understanding the concerns of heart specialists and joining PASCAR in his fight against CVD.

Prof Samuel Omokhodion, the then secretary-general, recalled the hard work of PASCAR’s founders and subsequent leaders through a brief history, and their merits for having created an organisation such as PASCAR, which has been integral to improving the cardiovascular health of the people of Africa over the last three decades, despite a difficult socio-economic and political context. The secretary-general also expanded on the challenges that the present and future generations face to carry on the charge of furthering the health of Africans.

President Macky Sall expressed his gratitude to the organisers of the meeting for having solicited him and said he was proud to be in the ‘heart of heart specialists’. He was fully convinced and would advocate everywhere that Africa must build protections against heart disease so as to stop hypertension and other cardiac risk factors from becoming established epidemics on the continent. He welcomed all the PASCAR delegates to Senegal, a welcome worthy of the legendary Senegalese ‘teranga’ (the Wolof word for hospitality is ‘teranga’ and it is so identified with the pride of Senegal that the national football team is known as the Lions of Teranga). About 700 delegates from English-, French- and Portuguese-speaking African countries and also from Europe and the USA attended the Dakar meeting.

## Workshops

Five workshops ocurred simultaneously on Wednesday morning and were very well attended. The workshop on interventional cardiology was hosted by the Pan-African Course on Interventional Cardiology (PAFCIC), (represented by Prof Habib Gamra, Tunisia), PASCAR (represented by Prof Bongani Mayosi, South Africa) and the American College of Cardiology/Association of Black Cardiologists (represented by Prof Ola Akinboboye, USA).

This workshop highlighted the growing incidence of ischaemic heart disease among Africans and the current difficulties in management, with very few acute cases benefiting from lytic therapy or primary coronary interventions. Some African countries such as Kenya have achieved significant progress in terms of acquiring invasive cardiac catheterisation laboratories, mainly in the private sector. Establishing a collaborative training and research programme between PAFCIC, PASCAR and ACC/ABC will help increase the state of readiness of cardiovascular practitioners to cope with the rising burden of disease.

The echocardiography, rhythmology and paramedical workshops were very practical (hands-on training) sessions and were well attended by delegates.

## Scientific sessions

## Obesity, diabetes and hypertension

Dr Andre Pascal Kengne, a distinguished Cameroon researcher from the Medical Research Council, South Africa stated that evidence has been accumulating on the importance of the rising burden of diabetes mellitus and obesity on the African continent. This has been at an increasingly higher pace than previously expected and than elsewhere in the world. He showed important differences in prevalence across countries and between rural and urban regions.^2^,^3^

Prof Said Norou Diop (Senegal) stated that the care for diabetes largely remains suboptimal in most countries, which are not adequately prepared to face the prevention and control of diabetes. The costs of caring for the condition pose a tremendous challenge to most local economies. Prof Jean Jacques Monsuez (France) described the higher prevalence of CVD among diabetic patients.

Prof Terrence Forester (Jamaica) showed that nutrition in early life influences the pathogenesis and prevention of cardiometabolic disease in Africans. The panel concluded that research is needed to contextualise the existing evidence for diabetes screening and prevention in African settings, and to better characterise the interaction of genetic and environmental factors on the occurrence of diabetes and obesity on the continent.

The rising incidence of obesity and diabetes in Africa parallels the incidence of hypertension. Prof Moustapha Saar (Senegal) presented hypertension as the dominant risk factor for CVD in Africa, with a prevalence ranging from 20 to 35% of the adult population, and this prevalence is anticipated to rise dramatically. Dr Daniel Lemogoum (Cameroon) said hypertension is currently poorly diagnosed, poorly treated and poorly controlled in Africa, exposing the African continent to the burden of serious adverse outcomes. He concluded that an updated but simplified Pan-African guideline for management of this disease is needed.^4^

Prof Jean Jacques Blacher (France) presented the current French and also European guidelines for treatment of hypertension. He insisted on the use of the triad combination of a diuretic, an inhibitor of the renin angiotensin aldosterone system and a calcium channel blocker whenever necessary to control resistant hypertension. The panel concluded that decisive actions are needed by African governments and policymakers to stop the negative health effects of uncontrolled hypertension.

## Stroke

Prof Mouhamadou Mansour Ndiaye (Senegal) gave an overview of the epidemiology and diagnosis of stroke. According to him, the burden of cerebrovascular disease manifests mainly as stroke caused by high blood pressure, and is now an established important cause of premature disability, morbidity and mortality in most regions of the continent.

Prof Ibrahima Diakhaté (Senegal) advocated for the increased availability of non-invasive imaging of stroke in most African centres. Prof Albertino Damasceno (Mozambique) presented the management strategies of stroke, which need to be effected through concerted implementation of several public health measures, primary and secondary prevention policies and costeffective treatment, running in parallel with and underpinned by coordinated research initiatives.^5^

## Heart failure and cardiomyopathies

The results of THESUS-HF, the African prospective registry of heart failure, which were published in 2012, were presented by Prof Albertino Damasceno (Mozambique).^6^ This study has provided several key insights into the epidemiology and prognosis of acute heart failure and cardiomyopathy on the continent, with hypertension emerging as the leading risk factor for heart failure, highlighting once more the need to place the treatment and control of hypertension in a central role for the global improvement of cardiovascular health in Africans.

Prof Abdoul Kane (Senegal) gave a comprehensive lecture on heart failure with preserved ejection fraction, with emphasis on new approaches to diagnosing the disease. Prof Karen Sliwa (South Africa) demonstrated that peripartum cardiomyopathy (PPCM) is one of the prevalent aetiologies of heart failure in women and is associated with adverse outcomes. She stated that the discovery of potential mechanistic pathways for cardiomyopathy is raising the promise of new interventions, such as the proposed role of bromocriptine in the treatment of this disease.^7^ Prof Jean Claude Daubert (France) gave a historical perspective of cardiac resynchronisation therapy for heart failure, with reference to its feasibility in Africa.

## Oral communications, posters and symposia

The Dakar meeting topped the number of abstracts ever submitted to a PASCAR meeting, with 48 oral communications and 147 posters presented. Three symposia were organised by pharmaceutical companies.

## Multicentric and collaborative research projects

Preliminary results of multicentric collaborative research projects being conducted on the continent were presented. Drs Friedrich Thienneman and Anastase Dzudie presented the Pan-African PUlmonary hypertension COhort study (PAPUCO) platform, which was launched at the PASCAR meeting in May 2011 in Kampala, Uganda. The study has recruited 132 patients with pulmonary hypertension. It is aimed to complete the recruitment of 200 patients with pulmonary hypertension by the end of August 2013, and six months of follow up will be completed by February 2014.

Dr Liesl Zuhlke (South Africa) presented preliminary results of the Global Rheumatic Heart Disease Registry (REMEDY),^8^ followed by Prof Samuel Kingue (Cameroon) for the Valvafric study, a registry on rheumatic heart disease (RHD). Both registries concluded that RHD is still a major disease on the continent and associated with poor outcomes, especially in the absence of cardiac surgery.

## Other sessions

The cardiac surgery and cardiac pacing sessions emphasised the need for training more specialised cardiologists and cardiac surgeons but also on the means to increase patient access to cardiac surgery and pacemakers. Sessions on vascular medicine as well as cardiac imaging were equally well attended and highlighted the low availability and affordability of other cardiac imaging, such as nuclear imaging, CT angiography and magnetic resonance imaging in African settings. Dr Ntobeko Ntusi from South Africa stated that while echocardiography will remain first line, other modalities do provide additional information, which affects management and henceforth will be made available in centres of excellence in Africa.

## General assembly meeting

The meeting was held on 17 May and started at 17:40 with the vice president (West), Prof Abdou Ba presiding. The secretarygeneral, Prof Omokhodion circulated the working document for the constitutional review, the suggested amendments were discussed by the assembly, and the views of the congress were adopted.

Ten distinguished members were honored for the PASCAR Award, given their significant contributions in the foundation and/or growing of PASCAR. They were Prof Ayodele O Falase (Nigeria), Prof Olufemi Jaiyesimi (Nigeria), Prof Papa Koate (Senegal), Prof WFT Muna (Cameroon), Prof H Ojiambo (Kenya), Prof OM Pobee (Ghana), Prof JK Manuwelle (Zimbabwe), Prof HS Badawi (Egypt), Prof Hippolyte Agboton (Benin) and Prof Peter Omollo Odhiambo (Kenya).

A new executive was elected under the leadership of Prof Bongani M Mayosi, who then outlined four priorities for the society over the next four years: (1) to establish a strong administrative and financial base, (2) to satisfy individual needs of members, especially in the area of training, (3) to boost research on the continent so that Africa can gain respect in the world in the area of cardiovascular disease, and (4) to reach out to North African societies. He also stressed the need to ensure that ministries of health adopt and implement the ‘10 Best Buys’ to combat heart disease, diabetes and stroke in Africa.^9^ The success or failure of PASCAR will be judged on the extent to which these goals are achieved over the next four years.

## Conclusion

This Dakar conference has once more confirmed the role of PASCAR as the premier umbrella association for national professional societies in cardiovascular medicine and surgery on the African continent. The election of a new and ambitious leadership demonstrated that PASCAR has turned the page of stagnation. It is now full of vitality and is poised to lead the continent, and be a leading force in area management of cardiovascular disease in the world during the third millennium.
